# Reply to Alsarwani, R.M. Comment on “Alghnam et al. The Association between Obesity and Chronic Conditions: Results from a Large Electronic Health Records System in Saudi Arabia. *Int. J. Environ. Res. Public Health* 2021, *18*, 12361”

**DOI:** 10.3390/ijerph19169848

**Published:** 2022-08-10

**Authors:** Suliman Alghnam, Saleh A. Alessy, Mohamed Bosaad, Sarah Alzahrani, Ibrahim Al Alwan, Ali Alqarni, Riyadh Alshammari, Mohammed Al Dubayee, Majid Alfadhel

**Affiliations:** 1 Population Health Section, King Abdullah International Medical Research Centre (KAIMRC), King Saud Bin Abdulaziz University for Health Sciences (KSAU-HS), Riyadh 11426, Saudi Arabia; 2 Public Health Department, College of Health Sciences, Saudi Electronic University, Riyadh 11673, Saudi Arabia; 3 Department of Epidemiology, School of Public Health, University of Pittsburg, Pittsburg, PA 15261, USA; 4 Division of Endocrinology, Department of Pediatrics, King Abdulaziz Medical City, King Abdullah Specialist Children’s Hospital, MNG-HA, Riyadh 11426, Saudi Arabia; 5 College of Medicine, King Saud Bin Abdulaziz University for Health Sciences, King Abdulaziz Medical City, MNG-HA, Riyadh 11426, Saudi Arabia; 6 King Abdullah International Medical Research Centre, King Saud Bin Abdulaziz University for Health Sciences (KSAU-HS), King Abdulaziz Medical City, MNG-HA, Alahsa 11426, Saudi Arabia; 7 School of Public Health, King Saud Bin Abdulaziz University for Health Sciences (KSAU-HS), Riyadh 11426, Saudi Arabia; 8 Medical Genomic Research Department, King Abdullah International Medical Research Centre, King Abdulaziz Medical City, MNG-HA, Riyadh 11426, Saudi Arabia; 9 Genetics and Precision Medicine Department (GPM), King Abdulaziz Medical City, King Abdullah Specialist Children’s Hospital, MNG-HA, Riyadh 11426, Saudi Arabia

We genuinely thank Dr. Alsarwani for his insights [1]. Our study is the first large analysis that has utilized electronic sources to examine significant population health predictors and outcomes [2]. Similar to most published studies, it has some strengths, limitations, and some potential bias which we have acknowledged. Our role as researchers is to determine truer estimates and reduce the magnitude of bias.

As for the first point about medications, some medications are indeed used to treat conditions other than diabetes. However, removing all patients with these medications would remove sizeable true diabatic patients and drastically underestimate the true prevalence. This is because ICD-10 coding is not mature enough to locally identify all patients with a specific disease. In addition, many patients come to clinics to treat one condition (i.e., asthma) while also being diabetics. The systems will not capture the primary coding of these patients because it was not the main reason the patients visited the hospital. From the most reliable national study conducted in 2013, we know that the national prevalence rates of diabetes and hypertension are 13.4% and 15.2%, respectively [3,4]. This estimate is about one decade old. We know that with increased risk factors, such as obesity and a sedentary lifestyle, diabetes and hypertension will likely increase in subsequent years. In fact, the recent PURE study, cited in our paper, indicated that the diabetes and hypertension prevalence rates were 25.1% and 30.3%, respectively, among older adults [5]. Finally, as part of the limitations, we have acknowledged that these estimates are based on hospital visits, which reflect those who sought medical treatment. Therefore, even if there was some overestimation of cases, it is likely to be minimal. We are currently working on a study to explore what percentage of diabetes is captured via all available sources such as ICD, medications, HA1C, or even using the progress note (written by the physician) alone. We hope that such a study will shed more light on the path to better capture all patients with a specific diagnosis with high sensitivity and specificity. 

As for the second point, it is unlikely that we missed those with type 1 diabetes because we included all those with a diabetes diagnosis. In fact, this was one of the limitations we stated in our study—we were unable to differentiate between type 1 and type 2 diabetes (page 8). 

As for the last point, this study examined the independent association between these variables and the outcome. Although many consider hypertension to be a confounder in obesity–diabetes associations, hypertension is likely also a mediator or a collider in that relationship. Therefore, adjusting for hypertension will bias that association [6].

## Data Availability

All data are available from the corresponding author upon reasonable request.
